# Attention Optimization Method for EEG via the TGAM

**DOI:** 10.1155/2020/6427305

**Published:** 2020-06-18

**Authors:** Yu Wu, Ning Xie

**Affiliations:** ^1^Glasgow College, University of Electronic Science and Technology of China, 611731, China; ^2^Center of Future Media, School of Computer Science and Engineering, University of Electronic Science and Technology of China, 611731, China

## Abstract

Since the 21st century, noninvasive brain-computer interface (BCI) has developed rapidly, and brain-computer devices have gradually moved from the laboratory to the mass market. Among them, the TGAM (ThinkGear Asic Module) and its encapsulate algorithm have been adopted by many research teams and faculty members around the world. However, due to the limited development cost, the effectiveness of the algorithm to calculate data is not satisfactory. This paper proposes an attention optimization algorithm based on the TGAM for EEG data feedback. Considering that the data output of the TGAM encapsulate algorithm fluctuates greatly, the delay is high and the accuracy is low. The experimental results demonstrated that our algorithm can optimize EEG data, so that with the same or even lower delay and without changing the encapsulate algorithm of the module itself, it can significantly improve the performance of attention data, greatly improve the stability and accuracy of data, and achieve better results in practical applications.

## 1. Introduction

In recent years, a control system based on related concepts such as human mind, thought, and consciousness has developed rapidly. The difference between this system and traditional computerized control systems is that it is based directly on the human brain, using electroencephalogram (EEG) as the basis for data analysis. This class is also known as brain-computer interface (BCI), in which the human brain controls the corresponding system [1].

EEG was first discovered by Richard in 1875 while studying the brains of exposed rabbits. In 1924, Hans published the first paper on scalp signal which can trace to EEG. Although electroencephalography has been found for more than a hundred years, in earlier studies, because the brain's thoughts, minds, and other activities typically produced signals with much lower amplitude than the EEG, most of the signals were submerged in spontaneous potentials [2]. Based on the original EEG alone, the researchers were unlikely to get “specific sensory and complex cognitive information” about the subject [3]. This is a major difficulty in EEG analysis.

## 2. Technical Background

Recently, the breakthrough of biotechnology and the rapid development of computer technology make the EEG and brain-computer interface, which is in the interdisciplinary field, develop rapidly. With the discovery of key characteristics of EEG such as event-related potential (ERP) [4, 5], P300 potential [6, 7] and contingent negative variation (CNV) [8], the technology gradually moved from ideal to reality. In 1999, *Nature* published the first paper on brain-computer interface. As a landmark achievement at that time, this paper showed that slow cortical potential (CSP) could realize the construction of brain-computer interface for spelling function [9]. In the 21st century, EEG technology has achieved the evolution from multinode invasive to multinode noninvasive and then to single-node noninvasive. EEG devices are moving from the laboratory to the mass market.

Single-node portable EEG equipment is a prevailing research field and has great potential in the future. At the core of many of these kinds of devices is the TGAM (ThinkGear Asic Module) and its encapsulate algorithm, with the difference being the Bluetooth version, the type of API, and how data is received by terminals. However, even though the single-node EEG device of the mind has been adopted by many research teams and faculty members around the world, due to the limited cost of technology development, the algorithm effect of calculating data is not satisfactory. There has been a lot of research using the TGAM, such as EEG control of wheelchairs to develop disability services [10], robotic hand controlled [11, 12], and brain training [13]. This device has a wide range of application fields. In addition to the traditional service equipment for the disabled, it has profound application potential and use value in the fields of game entertainment, VR environment, education [14], and human-computer interaction [15]. As early as 2014, there was a precedent for using the TGAM to test users' brain waves in games [16] which only detect the change of beta wave. However, the beta wave value given by the paper is quite different from the output value of the TGAM, so it can be seen that the modification module did not play a role in this experiment.

In particular, the application of EEG in VR is a hot topic in recent years. As early as 2012, the idea of building a hybrid system based on the advantages of EEG and VR appeared [17], and it can be used to optimize the sense of immersion in VR environment [18]. EEG must be an important part of the development of VR technology in the future.

The original TGAM was available in 2012, and despite some algorithm updates over the next eight years, the output data was still disappointing. The stability and accuracy of this module are far less than those of research-level equipment, and the existing open-source optimization method is quite feeble; whether official or private, there is no optimization algorithm for this widely used module, which is a pity for all the research projects using it.

## 3. The Design of Data Transmission via the TGAM

### 3.1. Data Transmission Method

The TGAM locks the wired transmission protocol from the inside and can only use Bluetooth for data transmission. The transmitted data is divided into large packets and small packets, which are all represented in a hexadecimal system in this paper. A packet, whether large or small, consists of three components: the packet header, the payload, and the checksum.

The small packets contain 8 bytes, one for every 2 ms. The big packets contain 36 bytes, with one big packet transferred after every 512 small packets. The data in the big packet (except for the data used for synchronization and checksum detection) is the result of calculating the raw wave value (raw data in short) in the previous 512 small packets and exporting it.

In the small packets, the value of the first two bytes are 0xAA for data synchronization, and the value of the third byte is 0x04, which means that the package is a small package with only 4 bytes of payload. The first three bytes are called packet headers, while data starts with the fourth byte (after which all data except the checksum is called Payload).

The fourth (0x80) and fifth (0x02) bytes represent the code and the length (2 bytes) of raw data, respectively. Sometimes, data like 0x80 and 0x02 are easily considered as packet header. However, it is clearly pointed out in the official manual that the checksum verifies all payloads, so 0x80 and 0x02 which are included in the checksum calculation formula are not packet header data, and the same is true for large packet data.

The two-byte length of the raw data is in the sixth (high eight bits) and seventh (low eight bits) bytes and is artificially set to transmit eight bits high and then eight bits low. Therefore, two bytes need to be spliced when the data is extracted, but the data needs to be further processed for the convenience of analysis. To obtain the new raw data with the absolute value within 32678, the data processing logic can be expressed as follows.

The last byte is the checksum, the module's own data detection mechanism. Add up all the payloads, invert, and then take the lower eight bits, so that the value and the eight-bit checksum are equal that means the packet is extracted correctly. That is Equation (1):,

(1) $$\begin{array}{c} \text{Checksum}=\text{Low}\_\text{eight}\_\text{bits }\left(\text{Sum}\_\text{of}\_\text{payload XOR }0\text{xFFFFFFFF}\right). \end{array}$$

The ratio of the number of packets transferred to the number of packets with checksum errors is the packet loss rate. The official manual states that the packet loss rate below 10% will not affect the final result [19].

The big packet also starts with two 0xAA for data synchronization. The third byte is 0x20 for 32 payloads. The first three bytes are packet headers. The payload starts from the fourth byte. The specific meaning of the data is not described here. It is important to note that the eight types of brainwave data transmitted in the packet are all divided into three bytes (the lowest bit is transmitted first) and need to be manually spliced. The transmission characteristics of the wave value can be found in Yin et al.'s research [20]; the corresponding description will not be elaborated in this paper.

### 3.2. Limitations of TGAM Encapsulate Algorithm

The encapsulate algorithm mainly has three limitations.

**Figure 1:**
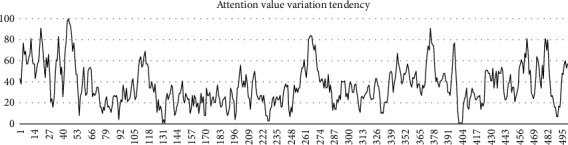
500 consecutive concentration values were randomly selected from the test data.

First of all, it may be the problem of development cost that TGAM, as a single-node EEG device, does not have good enough antinoise and data analysis step. No matter, in the experiment of this paper or the experimental data in some articles [21], the attention data calculated by the module's own algorithm is very unsatisfactory in practical application, with large fluctuations, high delay, and low accuracy (Figure 1). Some research projects have used large amounts of data to get baselines for attention and meditation to improve accuracy [22], but the results have been extremely limited.

Tests have shown that the aperiodical dips of attention value are caused by skin movements in the prefrontal, which is usually caused by the subjects blinking their eyes. In addition, subtle changes in the subjects' facial expressions can lead to irregular changes in EEG signals and sometimes even long periods of constant values, as well as spikes or drops in the difference of values above 50 (which has a range from 0 to 100).

Additionally, although there are 32 bytes of payloads in the big package (24 bytes of eight kinds of wave value data), it is difficult to make regular analysis because the hardware is a single-node sampling. In that case, the focus of the data analysis is mainly attention and meditation [23]. Both of these values are integers from 0 to 100, but the criterion of meditation is not as practical as attention in most cases, which is the reason why most research work using the TGAM will only analyze attention.

**Figure 2:**
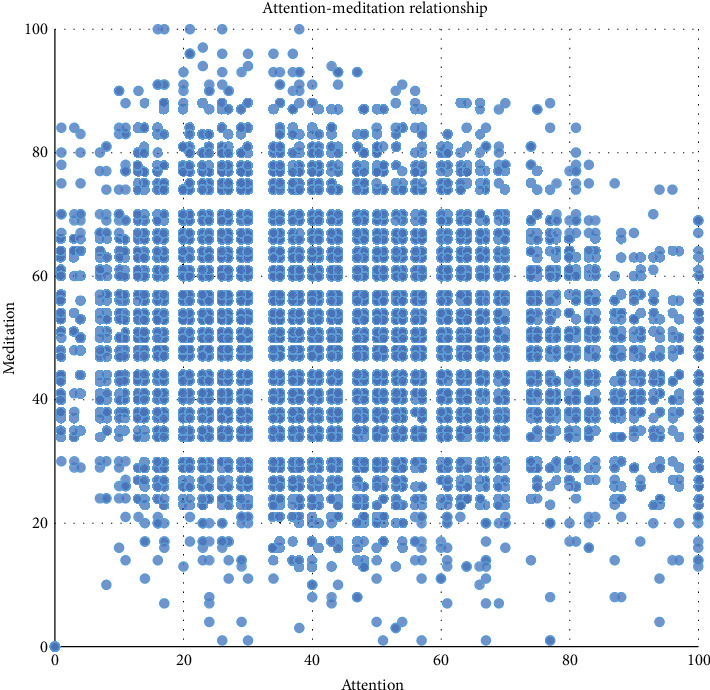
Encapsulate algorithm's output of attention-meditation relationship.

When we tried to find the relationship between attention and meditation, we found that these two values which are an output by the encapsulate algorithm of the TGAM were actually divided into many parts artificially (Figure 2), and this conclusion derives from a data set which contains over ten thousand samples from the experiment. Lots of meaningless empty value areas will increase the analysis cost. Specifically, the empty value areas are null region data points that do not appear in Figure 2. In other words, theoretically, there are 100^∗^100 = 10,000 kinds of data, because attention and meditation are all integers from 0 to 100. However, a large part of the data will not appear in experiments, and these areas that will not appear are called null value areas. The lack of stability in the data is partly due to the fact that 100 numbers divide the level of attention too much. Finding the attention-meditation relationship is not the first of its kind; a research had analyzed the relationship and used the wave value to make data correction [24]. But perhaps because of the small amount of data, the study did not find that the data had been artificially divided.

Finally, the encapsulate algorithm does not have error detection steps, even if there are a checksum and poor signal, which is used to verify the accuracy of the payload. In particular, the checksum is a standard for developers to check whether the data they receive from a module is correct. In other words, as long as the data is extracted correctly, the checksum will never report an error (our experiment can support this point). Poor signal is an integer between 0 and 200; 200 means that the developers cannot receive any signals by the wrong manner of wearing. This parameter is used to determine the quality of the device. Although the poor signal is 0, wrong data will still be output.

And there are three obvious problems with these two data (poor signal and checksum) that can be used for detection:

1. The output of a module does not consider checksum and poor signal. In other words, the data, such as attention, output of the encapsulate algorithm at each moment does not consider whether the checksum or poor signal at that moment is normal or not
2. After many tests, it can be seen that as long as the correct collection method is used, the checksum is almost always correct, which is of no essential help to data optimization. A total of 9698 groups of big packets were collected in one test, and none of them had incorrect checksum
3. Poor signal does not change continuously. In fact, there are only a few values such as 26, 51, 55, and 200, with a large number of null value areas. In addition, although the signal noise is zero in the case of data receiving, there are still unstable situations in Figure 1. It is obvious that this parameter is extremely limited

## 4. Attention Optimization Method

### 4.1. The Pipeline of the Proposed Optimization Method

**Figure 3:**
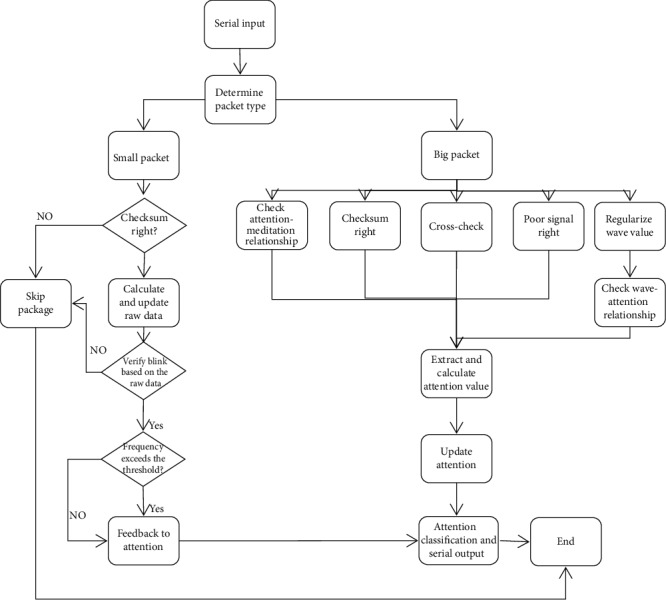
Flow diagram of optimization process.

Our algorithm optimizes two kinds of packages (big package and small package) to improve the performance of the data. The optimization process is summarized as follows (refer to Figure 3 for the overall optimization process):

1. Add checksum verification; that is, check whether the data value of the packet is consistent with the checksum and improves the accuracy of data collection
2. Cross-check the data with the previous big package. A big package with 36 bytes of data detects, but only attention and meditation will be detected
3. Because the eight kinds of wave value are a big integer with no practical significance which are defined personally, the result will be bad if dealt with directly. Consequently, use the Isolation Forest method to detect an outlier (abnormal data points) from a large number of wave value which is collected from experiment, and then find out the upper bound of eight wave values after the regularization. If at the same time there are three or more values beyond the upper bound, the packet at this moment will be judged as a wrong package. In addition, Isolation Forest is also used to find the relationship between the attention value and the meditation value and draw the decision boundary of the value of concentration on the meditation value
4. Because the blink signal will cause the instability of the raw data, which is manifested as a sudden drop in the attention value, the blink can be detected by the shake change of the small packets' data. When the user blinks, the algorithm will stabilize the value of attention from abnormal range to normal range. Additionally, there is a link between how often people blink and how focused they are [25, 26]. Therefore, a step was set to detect the blink frequency and to further optimize the data to improve the return value of attention
5. The output of attention is step by step, which means two constantly updated attention values are used to make the data with strong shake much smoother

There are three most important steps of this optimization algorithm, and the reasons and principle will be introduced in the next sections:

1. Time interval setting in big packet data extraction
2. Blink feedback mechanism
3. Output classification (removal of meaningless null)

### 4.2. Big Package Optimization via Isolation Forest

Before optimization, big packets' data extraction is the basis of the whole optimization algorithm system. Through experiments and investigations, it is found that the use of various versions of the official development kit or open-source code often encountered difficulties in extracting big packets' data. This is because when extracting, a code needs to wait a certain amount of time between each byte to be extracted. It is really important. Big packets lose data in a continuous read because of the defects of the original encapsulate algorithm. A large number of experiments have proved that if the pause time is not set, a large packet of data is successfully extracted every 3-4 seconds in general, but in theory, the large packet data is one for every 1026 ms. In order not to affect the accuracy of reading data, the pause time is smaller than the byte transmission time of large packets, and our algorithm takes 10 *μ*s.

After the checksum check is completed, cross-check with the previous packet is the second step of optimization. In a data set with more than 10,000 samples (under normal wearing conditions), no group of data has the same attention and meditation values as the previous group, and the two data are the same only when there is a problem in data collection. Therefore, cross-check can effectively eliminate incorrect data due to hardware contact or noise problems. After that, the poor signal at that moment will be extracted, which is located in the fifth byte of big packets. Poor signal can directly reflect the wearing quality.

After that, there are two boundary detection mechanisms. Our algorithm uses Isolation Forest to detect abnormal points and outline the decision boundary. Isolation Forest is an unsupervised anomaly detection method, which is suitable for continuous data. Isolation Forest uses a binary search tree structure (also known as “isolated tree”) to isolate samples and detect outliers through the isolation of sample points, which is different from the anomaly detection algorithm that expresses the degree of alienation between samples by quantitative indexes such as distance and density [27]. Because most of the attention values in the TGAM are in the correct range, the number of outliers is small and there is a significant difference from most samples. In that case, the isolation of sample points will cause the outliers to be isolated earlier; that is, the outliers will be closer to the root node, while the normal values will be further away from the root node. This is an important reason why this optimization algorithm uses Isolation Forest for upper- and lower-bound analysis.

**Figure 4:**
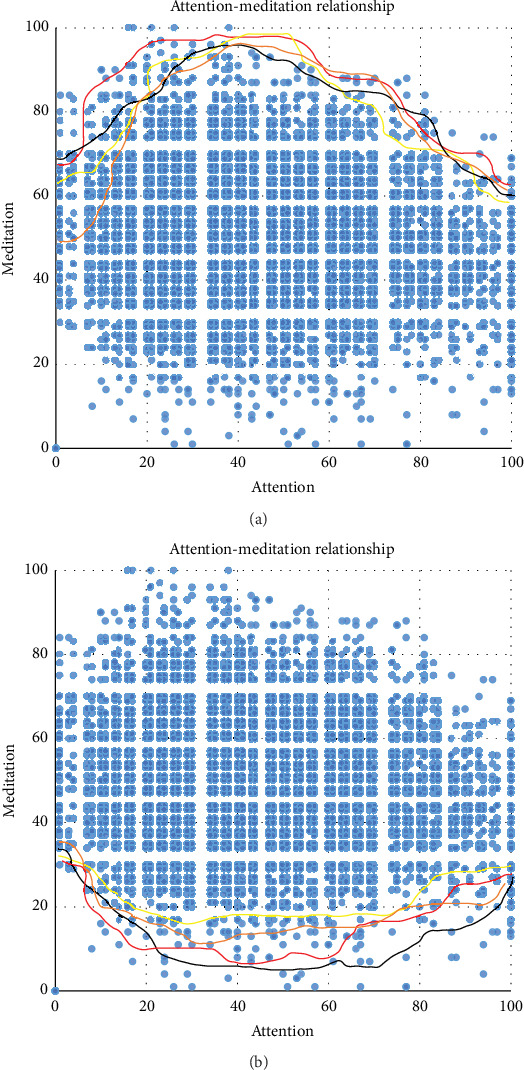
(a) Upper bound and (b) lower bound of attention-meditation relationship.

Finding the relationship between meditation and attention using the Isolation Forest method (Figure 4) is the first boundary detection mechanism. In the figure, there are four colored lines representing the boundary drawn by data from four different situations, and the final average of the four was taken. The boundary in the figure is the same as the trend of boundary which is drawn artificially, which is enough to prove the rigor and accuracy of this machine learning method.

Another boundary detection, specifically, is the detection of eight wave value data (respectively, Delta, Theta, LowAlpha, HighAlpha, LowBeta, HighBeta, LowGamma, mid-gamma); if more than three wave values fall outside the normal upper bound, it is judged as wrong data.

Before the decision, the wave value will be regularized, because it is a meaningless huge integer, which is too difficult to analyze. After trying standardization, normalization, regularization, and other methods and comparing with the untreated values, it is found that regularization is a method with obvious differentiation. Moreover, regularization will make the data fall between 0 and 1, and the upper and lower bounds of analysis are more convenient and effective. The results are as follows (Table 1).

The big packet optimization logic is shown below.

### 4.3. Small Package Optimization with Region Segmentation of Blink Frequency

The calculation method of the raw data in the small packets is mentioned in Section 3, and the range after parsing is between -32767 and 32768. Through the data set analysis, when the EEG signal is transmitted regularly, the raw data will be limited to ±550. The highest value is no more than 528, the lowest value is no less than -427, and most (over 95%) are within ±350.

**Figure 5:**
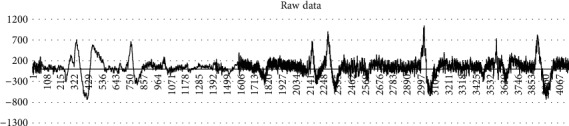
The raw data fluctuates with blink.

The raw data output from the device in eight seconds were randomly extracted (Figure 5) in a total of 4143 groups, and the obvious wave peaks were caused by the blink moment (7 times) of the tester. It can be seen that the raw data is quite stable when a blink does not happen, and the blink activity can be monitored only by detecting the fluctuations of the raw data.

In this algorithm, 1000 is set as the difference of the raw data to detect the blink signal, and the huge fluctuation of the raw data represents the blink movement of the user. The blink frequency can be calculated by calculating the time difference between each two blinks, and the attention value can be improved by calculating the specific frequency value, so as to further optimize the attention value. The method of judging the blink of the raw data can be expressed as follows.

The Blink_type above refers to the region where the blink frequency falls, which will be explained in more detail below.

Regarding the relationship between blinking and attention, Chen demonstrated that blinking not only affects the voltage of the cortex (that is, affects the EEG signal), but also negatively correlates the concentration with the blink frequency by comparing the blink frequency between the two groups of subjects during meditation and under normal conditions [26]. In the experiment, Chen concluded that relaxation and concentration resulted in a 3.8-6.0 times/min blink rate difference between the same subjects in different environments and at different times. In addition, Maffei and Angrilli have designed experiments that measure how quickly people blink when they complete tasks of different difficulty [25]. The harder and stranger the task, the less likely participants were to blink. This study also supports the interesting idea that when people focus on one thing, their concentration increases, but their reaction time to other things decreases [28]. The same conclusion emerged in a study of computer users who blinked significantly less when they looked at a screen [29].

To sum up, it is feasible to optimize the attention data by blinking.

The blink frequency was divided into five parts for analysis. The frequency selection was derived from the experimental results of Chen. The specific distinction is in Table 2.

The reason for setting too small and too large frequency as an invalid zone is to effectively avoid collection errors caused by hardware or wearing problems. The fluctuations in raw data caused by the blink itself can induce attention to plumb. In most cases, the blink will bring an 8-20 drop in the next attention value and after that, the next value will become regular. Therefore, even if the frequency is not in the focus zone, “blink compensation” needs to be set to keep the data close to the previous packet.

According to the data analysis and experimental feedback of blink moment, a relatively lower feedback value of 10 is used as the cardinal number of blink compensation. Additionally, frequency and maximum values are used to constitute the entire blink optimization system.

It should be noted that after the blink signal is detected, the system does not immediately modify the attention value but waits for the next big packet to arrive before making corrections. Doing this, relative to the direct update, fundamentally avoids the occurrence of the next abnormal attention value.

### 4.4. Tuning on the Attention Level

As mentioned in Section 4.1, there is no specific difference between each individual attention-meditation interval, and meaningless null value area will increase the analysis cost. The instability of the data is also partly due to the fact that 100 numbers divide the attention level too much. In the end, attention classification is a necessary step to optimize data, enhance accuracy, and reduce analysis costs.

It is different from the numerical classification in similar articles [30]. The algorithm classification system is essentially reflecting the TGAM itself way of classification. As shown in Figure 3, if not done classification in advance, cannot explain why the null value area so coincidence slices the image into many areas. Therefore, we remove the null value areas and divide the rectangular regions which are composed of blue data points in Figure 3. In this way, it can be divided into seven levels (Table 3) according to the abscissa (attention). The reliability of this attention level is proved in Section 5; it is crucial.

Among them, 0-4 and 98-100 actually have the attention value, but the algorithm still abandons them. For the former, this is because only 147 (1.52%) of the 9698 data sets are between 0 and 4, and more than half of them have nonzero poor signal. In addition, there are only two forms of these data: one is the continuous occurrence of multiple times with a huge difference with the data before and after. The other is the occurrence of only once with no connection with the data before and after as well. For the latter, only 100 will appear in the interval of 98-100, accounting for only 1.15% of the total number. The data will appear for several times in a row, and the difference with the previous data is more than 20 in most of times.

Combined with the fact that attention data rarely appear continuously and do not plunge or surge under regular circumstances, two areas can be abandoned.

## 5. Experimental Results

In order to prove the reliability of this optimization algorithm, the following experiment is designed.

Record the change of the attention value within a certain period of time in two specific application scenarios, and select game and study as test scenarios to correspond to the situations of intermittent concentration and high concentration, respectively. The time is about 20 minutes. Without any processing, about 1170 groups of attention value (one for every 1026 ms) will be collected, and 50 groups will be randomly extracted for data analysis.

Subjects should not have a history of mental illness or have taken psychiatric drugs and no history of epilepsy and other diseases that have a greater impact on brain fluctuations. On the night before the experiment, they were told to have a good rest and not to drink stimulants the day or the day before the experiment.

For the experimental environment, ensure that it is bright and quiet and try to choose a comfortable environment for the tested personnel. The volunteers were required to close their eyes and rest before starting the test. The EEG data reached a low noise and stable state, and then, the test of the corresponding scene was started at the command of the staff.

**Figure 6:**
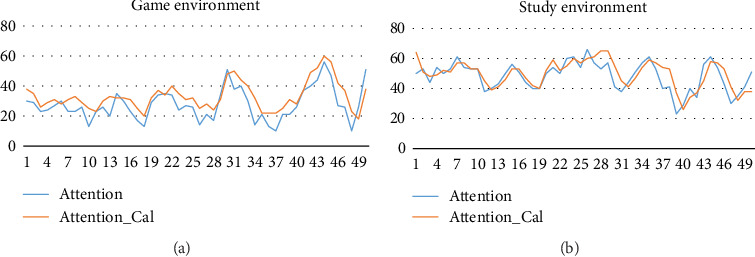
50 groups of attention value randomly extracted in (a) game and (b) study (scenarios after big packet optimization).

The data in Figure 6 is all after big packet optimization, where Attention_Cal is the result of small packet optimization (blink feedback). Obviously, even without small packet optimization, the attention value is still more stable and accurate than the data in Figure 1. Big packet optimization has eliminated most of the wrong data caused by hardware, microexpressions, and environment, while small packet optimization makes the change of attention more gradual and precise, with almost no abnormal sharp rise or plunge.

**Figure 7:**
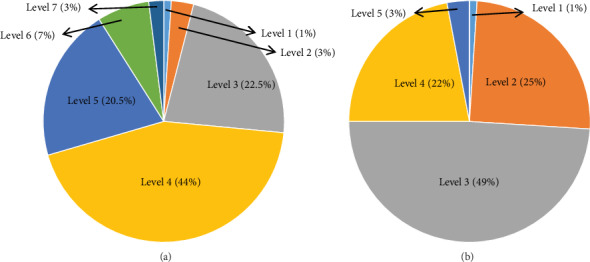
Proportion of the seven levels of attention in (a) study and (b) game.

Figure 7 shows the attention levels of situations. After grading, it can be seen clearly that the overall attention in study is much higher than that in game. And the grade distribution itself also conforms to the principle of normal distribution, so it can be seen that the results of this algorithm conform to the expected and subjective expectations of the volunteers. No change across two or more levels, or other abnormal changes, can be a stable reflection of the trend of attention value.

## 6. Conclusion

There are three most important steps of this algorithm, which are as follows: the time interval of the big packet data extraction setting, blink feedback mechanism, and classified output based on the removal of meaningless nulls.

The beneficial effect of this algorithm is to improve the performance of the attention value output from the TGAM without improving the real-time data transmission delay. The adverse effect of blink on EEG data is optimized and in turn feedbacks the degree of attention through the blink frequency, which greatly improves the stability and accuracy. Although the effect is significant, there are still two important problems.

First, EEG data is not as objective as traditional data. For example, in the magnitude of the velocity, we only need to calculate the ratio of displacement to time. However, as the objective reflection of thinking, the strong subjectivity and difficulty in describing thinking doomed the researchers to set subjective consciousness as the benchmark and create the analysis method of objective EEG data. Since the discovery of EEG in the 20th century, there has been a contradiction between objective data and subjective consciousness. The purpose of analyzing objective data is to reflect subjective consciousness, but is the existing objective data reliable enough to reflect real subjective consciousness? In the process of compiling and improving this algorithm, although the data optimization model has been fitted to the subjective consciousness of the testers as much as possible, the effect is not 100% satisfactory. This is not only a problem of this optimization algorithm but also a common problem of all EEG devices.

Second, because the encapsulate algorithm of the module is unknown, wave value analysis is almost impossible to achieve. Although we have tried to find the relationship between the eight wave value data and the attention, meditation, and raw data, we have failed in the case that the raw data analysis method is sealed. The TGAM itself is a product for developers, with more open-source data and more flexibility than a purely commercial EEG device. We believe that if the raw data analysis algorithm can also be open source, our optimization can be further improved to get better results.

Finally, the optimization algorithm is the improvement of software. In the field of electronics, the software and hardware should be combined to play the most important role. However, in the case of limited hardware conditions, this optimization algorithm also has certain practicability and promotion value.

## 7. Future Work

No matter how good the algorithm is, it needs to be verified by practical application [31], and it should be improved and perfected continuously in this process. So our next step is to explore the possibilities of our algorithm in application scenarios that require EEG analysis. Especially in VR applications, we believe that EEG analysis will make a difference.

EEG and BCI technologies are beginning to take off, and it is believed that such research will yield more brilliant results.

Furthermore, we would like to apply EEG into a more realistic practical research framework with crossmodal [32, 33] and multitask [34] in action recognition [31] and HCI in VR [35].

## Figures and Tables

**Algorithm 1:**
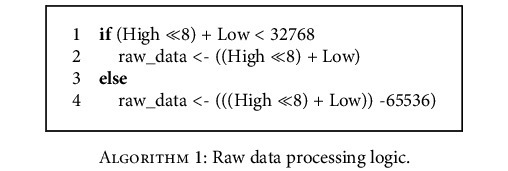
Raw data processing logic.

**Algorithm 2:**
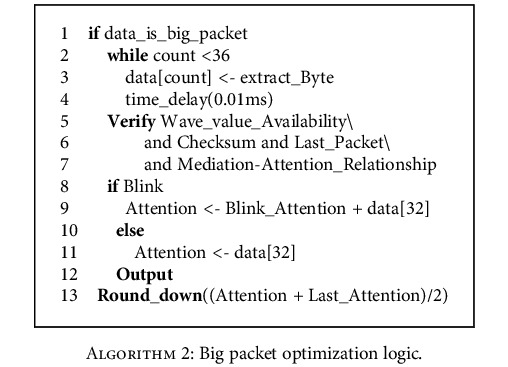
Big packet optimization logic.

**Algorithm 3:**
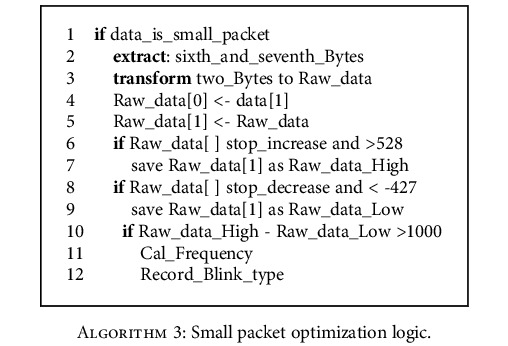
Small packet optimization logic.

**Table 1:** Threshold of wave value after regularizing.

Wave name	Delta	Theta	LowAlpha	HighAlpha	LowBeta	HighBeta	LowGamma	MidGamma
Threshold value (regularization)	0.635	0.610	0.640	0.600	0.615	0.605	0.620	0.630

**Table 2:** Region segmentation of blink frequency.

Frequency (second/once)	Region	Attention management
<2	Invalid area	None
2-4.81	Normal area	Plus 10
4.81-6.51	Focus area	Plus (10 + frequency × 0.8)
6.51-8.33	Highly focus area	Plus (10 + frequency × 1.5)
>8.33	Invalid area	None

**Table 3:** Attention classification method.

Attention level	Null value region	Boundary	Attention range	Meditation upper boundary	Meditation lower boundary
0	0-4	5-6	0-4		0
1	9, 12, 15	18-19	7-8	74	28
			10-11	77	26
			13-14	83	23
			16-17	87	19
2	22, 25, 28	31-33	20-21	90	16
			23-24	93	14
			26-27	97	11
			29-30	97	10
3	36, 39, 42	45-46	34-35	97	10
			37-38	97	13
			40-41	94	10
			43-44	94	10
4	49, 52, 55	58-59	47-48	91	10
			50-51	88	10
			53-54	88	10
			56-57	87	10
5	62, 65, 68	71-73	60-61	84	10
			63-64	84	10
			66-67	81	10
			69-70	81	16
6	76, 79, 82	85-86	74-75	78	20
			77-78	78	20
			80-81	75	24
			83-84	69	27
7	89, 92, 95	98-100	87-88	69	23
			90-91	67	27
			93-94	64	29
			96-97	58	33

## Data Availability

Please contact us by email if any data used in the manuscript is demanded.
